# Further Experience with Doyen's Cancer Serum

**Published:** 1906-01-20

**Authors:** 


### FURTHER EXPERIENCE WITH DOYEN'S CANCER SERUM.

Although this serum came upon the stage of therapeutics with a flourish of trumpets transcending the custom even of cures for cancer, there seems to be every likelihood that, like its horde of predecessors, Doyen's serum will be written down a failure. Dr. Alexis Thomson[1] has just published the results attending the use of this serum in the treatment of inoperable carcinoma. These results are not encouraging, in spite of the fact that one of the patients was seen by Dr. Germain See, Doyen's assistant, and pronounced by him to be a suitable case for the employment of the serum, while all the cases were treated by Dr. Barbour Simpson, who carried out the injections on the lines formulated by Doyen.

The first patient was a woman of 57, the subject of an extensive recurrence after an operation for the removal of a scirrhous carcinoma of the breast. The breast and axillary contents had been removed seven months before, while two months later the clavicle had been resected for the removal of infected glands from the posterior triangle of the neck. At the time when the serum treatment was begun she presented a combination of cancerous infiltration and of nodules in the vicinity of the scar. She suffered a good deal of pain, but her general health was good, and there was no evidence of dissemination of the growth in the viscera. In the course of a month ten injections were administered. These resulted in several instances in a considerable reaction evidenced by rise of temperature, but there was no improvement in the cancerous condition; the nodules in the skin increased in size so as to become almost confluent, while the pain in the arm became so severe as to demand control by hypodermic injections of morphine. Her general health became rapidly worse, and she died without any vestige of improvement. Cancerous nodules in the skin were submitted to microscopic examination both before and after the commencement of the serum treatment, but there was no evidence of any change in the histology of the growth as a result of the treatment. "Those who watched throughout the progress of this case," says Dr. Alexis Thomson, "were unable to observe any benefit which could be traced to the employment of the serum, and from the patient's point of view this added to the distress and failure of health incident to the disease."

The second case was precisely similar, both in the matter of the age and sex of the patient, and in the nature of the lesion. The body of the breast had been removed three and a half years before. The recurrent disease consisted of an ulcerating tumour below the scar of the former operation, together with a few nodules in the skin, and a large mass of glands in the axilla. The patient was a robust and well-preserved woman, apparently in the enjoyment of perfect health. This case fulfilled the conditions laid down by Doyen for successful treatment by means of his serum; all the visible disease could be removed by operation, and there was no evidence of dissemination of the disease beyond the area of its origin; at the same time, without the aid of the serum, operation would almost inevitably be followed by recurrence. The usual course of injections was given before the operation, and it was believed that the ulcer assumed a somewhat more healthy appearance; all the visible disease was then removed, and proven by miscroscopic examination to be a carcinoma. A further course of injections was given after the operation, and the patient returned home apparently in the best of health and without any visible signs of malignant disease. But in two months fresh nodules made their appearance in the vicinity of the scar; the disease progressed slowly, and the patient died within seven months of the second operation.

The two remaining cases given by Dr. Thomson are not of so much importance as those above recounted, for one of them was an epithelioma of the lower jaw, a type apparently which Doyen himself admits to be beyond the therapeutic aid of his serum; while the other was an instance of non-malignant ulcerating papilloma, thought at the time to be squamous celled carcinoma, though subsequent investigation negatived this belief. It is interesting to note that the reactions which followed the injections of serum in this case were quite as marked as those occurring in the two cases of mammary carcinoma detailed above.

Dr. Thomson observes that he has recorded these cases not because they, considered alone, afford any grounds for deciding upon the merits or demerits of the treatment, but because he wishes to assist in the accumulation of cases illustrating the effect of the serum. This is a perfectly correct and scientific attitude, but it cannot be said that the evidence so far forthcoming from independent sources is much in favour of Dr. Doyen's serum.

[1] Edin. Med. Jour., Jan. 1906.

